# Parental psychological control and children’s self-esteem: A longitudinal investigation in children with and without oppositional defiant problems

**DOI:** 10.1186/s13034-024-00740-0

**Published:** 2024-04-29

**Authors:** Yixin Tang, Sheida Novin, Xiuyun Lin, Andrik Becht, Sander Thomaes

**Affiliations:** 1 Department of Development Psychology, Utrecht University, Utrecht, The Netherlands; 2 Institute of Developmental Psychology, Faculty of Psychology, Beijing Normal University, Beijing, China; 3 Department of Education and Pedagogy, Utrecht University, Utrecht, The Netherlands

**Keywords:** Self-esteem, Maternal psychological control, Paternal psychological control, Oppositional defiant problems, Longitudinal study, China

## Abstract

Oppositional defiant problems are among the most prevalent psychological problems among children and adolescents from China and across the world. Still little is understood about how self-esteem, in conjunction with parenting experiences, develops in children with oppositional defiant problems. We addressed this gap of knowledge in a two-year longitudinal study. Specifically, we explored how parental psychological control predicts children’s self-esteem levels over time, and in turn, how children’s self-esteem levels predict parental psychological control. We collected data in Chinese children (ages 8 to 13 at T1) with (*N* = 224) and without (*N* = 217) oppositional defiant problems, and tested three-wave cross-lagged panel models. Multigroup analyses showed that the associations between parental psychological control and children’s self-esteem were the same for children with and without oppositional defiant problems. Results for the total sample revealed bi-directional associations between maternal psychological control and children’s self-esteem. Children who perceived more psychological control from their mothers were likely to exhibit lower self-esteem over time, and vice versa, children with lower self-esteem were likely to perceive more maternal psychological control over time. Conversely, a unidirectional paternal effect was observed in father-child dyads. Our findings help understand the parent–child dynamics that shape the psychological development of children with oppositional defiant problems.

**Supplementary Information:**

The online version contains supplementary material available at 10.1186/s13034-024-00740-0.

## Introduction

Oppositional defiant problems are prevalent among children and adolescents from China and across the world [1–3]. These problems are characterized by a recurrent pattern of oppositional behaviors toward authority figures, including argumentative, defiant, and vindictive behaviors [4–6]. Children with oppositional defiant problems are prone to experience anger, irritability, and resentfulness [7]. Still little is known about how children with oppositional defiant problems perceive and evaluate themselves. Given that these children often experience difficulty in their relationships with parents [8, 9], we longitudinally examined the self-esteem development of children with (vs. without) oppositional defiant problems as a function of a parenting practice that is intrusive, autonomy-limiting, and potentially harmful—i.e., parental psychological control. As the association between parenting and child functioning is inherently bidirectional and reciprocal [10], we also explored if children’s self-esteem reciprocally predicts parental psychological control. We examined these associations in the cultural context of China. Chinese cultural values emphasize the importance of social conformity, which may have ramifications for the psychological development—including self-esteem development—of children with oppositional defiant problems.

### Oppositional defiant problems and self esteem

Self-esteem, defined as an individual’s subjective evaluation of one’s worth as a person, is a key indicator of children’s psychological well-being [11, 12]. Children with oppositional defiant problems may be at risk for experiencing low levels of self-esteem. Theoretically, it is possible that these children use externalizing, oppositional defiant behaviors as a coping strategy to protect themselves against negative self-feelings associated with their interpersonal difficulties [13]. Indeed, a recent study conducted in community and clinical samples of children with oppositional defiant problems found that lower levels of self-esteem were associated with elevated behavior problems ([14], but see [15]). The social or psychological factors that account for this putative link, however, are still unknown. Why are children with oppositional defiant problems at risk for experiencing low self-esteem? One contributing factor may be the socialization practices of parents, who play a central role in shaping children’s self-esteem [16, 17]. One parenting practice that can be consequential for children’s self-esteem, and perhaps especially so in children with oppositional defiant problems, is parental psychological control [18, 19].

### From parental psychological control to self-esteem

Parental psychological control encompasses a set of practices that parents may use to control their children’s thoughts, feelings, and behaviors [20]. Examples include guilt induction, shaming, love withdrawal, and invalidation of children’s thoughts and feelings [21]. These practices are often intrusive and manipulative [22]. They can also interfere with children’s basic need for autonomy, and potentially limit children’s opportunities to form a secure, stable, and positive sense of self [19, 20, 23].

Indeed, cross-sectional and longitudinal studies have consistently revealed that children who perceive their parents as psychologically controlling also tend to report lower or decreasing levels of self-esteem (e.g., [24–26]). These effects have been found in children growing up in both Western and Eastern cultures. For instance, one study showed that parental psychological control predicts declines in self-esteem six months later, in samples of early adolescents from both the US and China [27]. Another study found that parental psychological control predicts declines in self-esteem roughly 18 months later, again in early adolescents from both the US and China [28]. In the latter study, Chinese children consistently reported lower self-esteem than their American counterparts, which the authors attributed to the elevated levels of parental psychological control that Chinese children reported. Hence, the available evidence is consistent with the view that parental psychological control practices may compromise the development of healthy self-esteem in children.

Notably, these studies were conducted in samples of typically developing children. Psychological theory and empirical research suggest that the effects of parental psychological control on children’s self-esteem may be magnified in children with oppositional defiant problems. Specifically, children with oppositional defiant problems often resist authority-imposed rules and expectations (e.g., from parents, teachers) and tend to prioritize their personal goals [29]. Such defiant behavior may reflect a heightened need for autonomy—a need that is thwarted when parents engage in psychologically controlling behavior [30–33]. Indeed, children with heightened need for autonomy are relatively sensitive to attempts to influence or control them [34, 35]. Therefore, parental psychological control may be particularly detrimental for the self-esteem development of children with oppositional defiant problems, as their self-esteem may be closely tied to the fulfillment of their autonomy needs [36].

### From self-esteem to parental psychological control

Of course, children are not just passive recipients of parenting. Rather, as transactional theory articulates, the association between parenting and child functioning is inherently bidirectional and reciprocal [10]. From this perspective, it is plausible that children’s self-esteem is not only influenced by parental psychological control (i.e., a ‘‘parent effect’’) but, vice versa, children’s self-esteem may also influence parental psychological control (i.e., a ‘‘child effect”; [37]). We know of no prior work that has tested this child effect. The present study is a first attempt to explore the potential effect of children’s self-esteem on their parents’ psychological control.

While direct evidence exploring the influence of children’s self-esteem on parental psychological control is lacking, research from neighboring fields is informative. Previous research has shown that parents who perceive some form of vulnerability in their children may be inclined, perhaps in an effort to help their children, to engage in controlling behaviors that inadvertently violate the autonomy-connectedness balance [22]. For example, research has shown that parents who believe their children cannot adequately regulate their emotions on their own, are inclined to use more psychologically controlling practices [38]. Furthermore, longitudinal studies have demonstrated that children’s internalizing symptoms, such as depression and anxiety, predict increases in perceived parental psychological control over time [39, 40]. While low self-esteem is distinct from these internalizing symptoms, it may similarly signal vulnerability to parents. Therefore, parents may resort to psychologically controlling practices in response to children’s low self-esteem, thus maintaining children’s dependence on them [41]. In summary, we propose that the link between parental psychological control and children’s self-esteem may be reciprocal and mutually reinforcing.

This negative reciprocity may especially be the case for children with oppositional defiant problems. Research has shown that parents sometimes engage in psychologically controlling behaviors to help their children to meet societal norms and expectations [22, 42]. Thus, when children experience both low self-esteem and oppositional-defiant problem behavior, parents might be especially inclined to be psychologically controlling. Social coercion theory [43] suggests that children with oppositional defiant problems are at risk of becoming trapped in negative reciprocity between escalating child behavior problems and problematic parenting behaviors. Accordingly, children with oppositional defiant problems might be at heightened risk of being impacted by negative reciprocity between parental psychological control and their own low self-esteem.

### Self-esteem and parental psychological control reciprocity in China

We test these dynamics between parental psychological control and children’s self-esteem in China. From a cultural comparison perspective, parenting practices in China tend to be relatively hierarchical and disciplinarian [44, 45]. Rooted in Confucianism, these parenting practices are shaped by the concept of “guan,” which reflects parents’ intensive monitoring, regulation, and involvement in their children’s lives to help them adhere to societal standards [28, 46, 47]. Accordingly, compared to their Western counterparts, Chinese parents tend to show relatively high levels of parental psychological control [1, 2, 28, 44]. We propose that, in China, there is increased tension between the norm for children to conform to societal standards and the behavioral difficulties of children with oppositional defiant problems. These problems can be challenging for parents in every cultural context, but possibly especially so in a cultural context that emphasizes social harmony and conformity. Chinese parents might thus be prone to exert psychological control to guide or discipline their children with oppositional defiant problems to conform to societal expectations, perhaps especially when these children also experience low self-esteem.

### The present study

We conducted a 2-year, 3-wave longitudinal study in China to examine the bidirectional associations between parental psychological control and child self-esteem, comparing children with and without oppositional defiant problems. We chose to focus on children in middle and late childhood (ages 8 to 13), as this is a time when individual differences in self-esteem emerge and parents exert strong influence on how children view themselves [11, 16, 17]. We hypothesized that higher levels of parental psychological control would predict lower levels of children’s self-esteem over time, and vice versa. Furthermore, we hypothesized that both the “parent effect” (i.e., parental psychological control predicting children’s self-esteem) and the ‘‘child effect’’ (i.e., children’s self-esteem predicting parental psychological control) would be magnified in children with (vs. without) oppositional defiant problems.

As stressed by family systems theory [48] and social role theory [49], mothers and fathers can contribute differently to parent–child interactions and child development. In China, mothers tend to be more involved than fathers in daily childcare and in addressing emotional needs of their children [50–52]. Fathers tend to engage less frequently in emotional interactions and may be more likely to contribute to child development through play and discipline [53, 54]. Prior research has focused on the consequences of either maternal psychological control or aggregated maternal and paternal psychological control—thus, our understanding of how paternal psychological control, specifically, influences children’s self-esteem development is limited [40, 55]. Our study adopts an exploratory approach to examine associations between parental psychological control and child self-esteem for mothers and fathers separately.

## Method

### Participants and procedure

Data were derived from the longitudinal study “Family Risk and Protective Factors of Oppositional Defiant Disorder (ODD) in China” [9, 57] Children were recruited from 14 primary schools located in the North, East, and Southwest of mainland China. These regions were chosen to represent a spectrum of socio-economically developed and less developed regions within China. First, we obtained the consent of the school principals and school psychologists. Next, we requested the school psychologists to distribute research invitations and informed consent forms to class master teachers. A total of 187 class master teachers signed informed consent forms and agreed to participate in our study with their classes.

To identify children with oppositional defiant problems, we used a two-step procedure. First, class master teachers nominated children who had shown at least four ODD symptoms over the last six months, from a total of eight ODD symptoms listed in the Diagnostic and Statistical Manual of Mental Disorders (DSM‐IV‐TR; [5]). We chose this first step in the procedure for the following reasons: (a) in China, class master teachers know their students well—they have been able to observe their students’ behavior in school for a long time, given that they typically teach the same class throughout all primary school years; and (b) previous research has shown that teachers are relatively accurate in their reports of child ODD symptoms [56–58]. A total of 360 children, 4.5% out of the total of 7966 children who were enrolled in the participating schools, were initially nominated as potentially having oppositional defiant problems. Second, two licensed clinical psychologists from the research team conducted semi-structured interviews with the class master teachers to confirm children’s ODD symptoms. While this approach did not allow for establishing clinical diagnosis, the clinical psychologists did make use of the DSM-IV-TR guidelines to confirm children’s oppositional defiant problems: (a) the child showed four or more ODD symptoms; (b) these symptoms lasted for six months or longer, and (c) the child exhibited significant impairment in psychosocial functioning. A total of 305 children, 3.8% of the total pupil population in the participating schools, were confirmed to have oppositional defiant problems. Next, invitation and informed consent letters were sent to these children’s parents. In total, 282 parent–child dyads agreed to participate in the study (92% participation rate), and 256 children (from 156 classrooms) completed the first assessment (T1). A comparison group of children without oppositional defiant problems was also recruited from the same classes, and with similar procedures. The inclusion criteria for this group were the following: the child exhibited (a) no or less than four oppositional defiant symptoms over the last 6 months; and (b) no mental disorder or physical disability. We randomly selected a group of children without oppositional defiant problems (similar in size to the group of children with oppositional defiant problems), from the same 156 classrooms. A total of 265 children completed the first assessment (T1).

For the purposes of the present study, we excluded the youngest children (i.e., 6- and 7-year-olds) from our sample, because it is only from about age 8 that meaningful individual differences in children’s self-esteem emerge [11]. As such, the final sample of children with oppositional defiant problems included 224 children (70.9% boys, 1 unspecified; *M*_ageT1_ = 9.92, *SD* = 1.50). Of them, 78.97% were the only child in their family. More than half of the mothers (53.55%) and fathers (59.24%) had completed junior college or higher levels of education. Families came from diverse socioeconomic backgrounds, 54.25% of families had a monthly income over 5000 Chinese RMB (the average monthly income for Chinese urban families at the time of data collection was about 5485 Chinese RMB; National Health & Family Planning Commission of the PRC, 2015). The final sample of children without oppositional defiant problems included 217 children (53.5% boys, *M*_ageT1_ = 9.60, *SD* = 1.42). Of them, 80.47% were the only child in their family. More than half of mothers (64.11%) and fathers (69.67%) had completed junior college or higher levels of education. Families came from diverse but on average somewhat better-off socioeconomic backgrounds, 72.60% families had a monthly income over 5000 Chinese RMB. Additionally, six children had missing ODD symptom data. We included available data from these six children in the total sample analyses but excluded them from the group comparison analyses.

The research protocol was approved by the Institutional Review Board of Beijing Normal University. Children completed questionnaires in class during regular school hours, assisted by trained research assistants. Participating families were offered the opportunity for consultation with psychiatrists at a local hospital, or with psychological counselors and family therapists affiliated with the research group. Approximately 1 year (T2) and 2 years (T3) later, children completed the same questionnaires in their classes again. Each participant (i.e., parents, children, and teachers) received a small payment (equivalent to $8) as compensation for their participation in the study.

### Measures

#### Child self-esteem

Children reported their global sense of worth as a person using the Rosenberg Self-Esteem Scale (RSES; [59, 60]). A sample item includes “Overall, I am satisfied with myself”. The Chinese version of the RSES consists of 9 rather than 10 items, because one of the original items (“I wish I could have more respect for myself”) cannot be translated well [61]. Participants rated items using a 4-point Likert scale (1 = “strongly disagree”, 4 = “strongly agree”. Negatively worded items were reverse scored, so that higher mean scores indicate higher self-esteem. The internal consistency of the RSES was good for both children with and without oppositional defiant problems from T1-T3 (αs ranged from 0.82 to 0.84, and from 0.81 to 0.84, respectively).

#### Parental psychological control

Children reported their perceived maternal and paternal psychological control using the Chinese Maternal/Paternal Psychological Control Scale [24]. A sample item includes “My mother/father always wants to change my views to fit his/her standards”.^1^ Participants rated items using a 4-point Likert scale (1 = “strongly disagree” to 4 = “strongly agree”). The internal consistency was good for both children with and without oppositional defiant problems from T1-T3 (for the Maternal Psychological Control Scale, *α*s ranged from 0.84 to 0.90, and from 0.73 to 0.88, respectively; for the Paternal Psychological Control Scale, *α*s ranged from 0.83 to 0.90, and from 0.74 to 0.88, respectively).

### Data analytic plan

We conducted all analyses in R using the Lavaan package [62]. To test bi-directional associations between parental psychological control and children’s self-esteem, we conducted three-wave Cross-Lagged Panel Models (CLPM) separately for mothers and fathers. We chose CLPM as our primary analytical approach due to its robustness in identifying between-person effects [63], which are central to our research question pertaining to prospective between-person associations between parental psychological control and children’s self-esteem, and how such associations may differ between children with and without oppositional defiant problems. We additionally used Random Intercept Cross-lagged Panel Models (RI-CLPM) to explore potential within-person associations between parental psychological control and children’s self-esteem [64]. RI-CLPM can estimate within-person associations by accounting for the overall stability of construct relationships at the between-person level, further enhancing our understanding of how parental psychological control and children’s self-esteem are associated.

In CLPM, concurrent associations (i.e., the correlation between maternal/paternal psychological control and child self-esteem) were included at each measurement time. Autoregressive paths (i.e., the predictive effect of a variable on itself) were included for both maternal/paternal psychological control and child self-esteem. Cross-lagged paths (i.e., the predictive effect of one variable on another variable at the next wave) were included from maternal/paternal psychological control to child self-esteem and from child self-esteem to maternal/paternal psychological control. For the sake of parsimony, we constrained autoregressive and cross-lagged paths to be equal from T1 to T2 and from T2 to T3. We used Chi-square difference testing to examine whether the model fit differed significantly between the unconstrained and the constrained model. If the constrained model did not fit significantly worse, we interpreted findings from the constrained model. ﻿Based on the CLPM, the RI-CLPM incorporated random intercepts to account for the stable individual differences, with the autoregressive and cross-lagged effects representing within-person associations.

To test potential differences in reciprocal associations for children with and without oppositional defiant problems, we conducted multigroup analyses. The two-group analyses consisted of several nested models, including a baseline model, a fully constrained model, and two parsimonious models. The baseline model freely estimated the autoregressive and cross-lagged paths without any between-group equality constraints. The fully constrained model constrained all autoregressive and cross-lagged paths to be equal across groups. The two parsimonious models include the “equal parental effect” model with parental effect paths constrained to be equal between the two groups, and the “equal child effect” model with child effect paths constrained to be equal between the two groups, while the other paths are freely estimated.

We examined model fit using the Tucker-Lewis Index (TLI), Comparative Fit Index (CFI), the Root Mean Square Error of Approximation (RMSEA), and the Standardized Root Mean Squared Residual (SRMR). For the SRMR and RMSEA, values below 0.08 indicate adequate fit and values below 0.06 indicate excellent fit. For the CFI and TLI, values above 0.90 indicate acceptable fit and values above 0.95 indicate excellent fit [65]. We used maximum likelihood estimation with robust standard errors (MLR) to account for non-normal distributions, and Satorra–Bentler scaled χ^2^-difference test to compare model fit.

### Power analysis

We used data from a larger longitudinal study for which the sampling approach was to include as many children from the involved schools as possible. We conducted a post hoc power analysis to determine the actual power we had when running our models [66]. We determined the actual power with the available sample size to detect misspecifications of a model corresponding to RMSEA = 0.08 for an alpha error of 0.05. Additionally, we calculated power for each estimated pathway in our models using a Monte Carlo simulation [67]. To do this, we first re-estimated our models in Mplus 8.0 [68] and saved the starting values. Then, we ran a Monte Carlo simulation for each model, using the starting values as input. We simulated the achieved power with the actual sample size as well as with increasingly large samples up to *n* = 10,000, to test the required sample size for sufficient power for each estimated pathway.

### Transparency and openness

The dataset and R scripts for the analyses are available on the OSF page for our study (https://osf.io/5wqrd/). We followed Journal Article Reporting Standards (JARS, [69]), and report all data exclusions and all measures in the study. Our study was not pre-registered.

## Results

### Descriptive statistics, correlations, and preliminary analyses

Sample attrition over time was relatively small. Of the 447 children who participated at T1, 414 (92.62%) children participated at T2, and 331 (74.05%) children participated at T3. A multivariate analysis of variance (MANOVA) found no differences between children who dropped out of the study and those who did not on any of the study variables (i.e. child self-esteem, maternal and paternal psychological control) at T1, *F*(3, 402) = 0.24, *p* = 0.869, η^2^ = 0.002. Missing data on the study variables ranged from 2.24% to 29.31% across all time points. Little’s missing completely at random (MCAR) test showed that the pattern of missing values was random, χ^2^/*df* = 1.25, indicating that it is unlikely that our findings were biased due to missing values. We therefore decided to make use of all available data in our analyses. Missing data were handled using full information maximum likelihood.

We performed preliminary analyses to evaluate descriptive statistics for children’s gender, age, self-esteem, and parental psychological control (see Table 1). Children with oppositional defiant problems were more likely to be boy, χ^2^ = 13.44, *p* < 0.001, and older, *t* = −2.59, *p* = 0.010, than were children without oppositional defiant problems. At all three time points, children with oppositional defiant problems reported lower levels of self-esteem, *p*s < 0.001, *d*s < −0.25, higher levels of maternal psychological control, *p*s < 0.001, *d*s > 0.25, and paternal psychological control, *p*s < 0.001, *d*s > 0.19, than children without oppositional defiant problems. We conducted one additional, exploratory analysis to test how children’s self-esteem levels changed over time in both groups (see Additional file 1: S1). This analysis showed that children with oppositional defiant problems reported lower but slightly increasing levels of self-esteem over the study period. No such self-esteem increase was observed for children without oppositional defiant problems, although the slopes of self-esteem change did not significantly differ between the two groups.

**Table 1:** Descriptive statistics for both samples

	Children without oppositional defiant problems % or *M* (*SD*)	Children with oppositional defiant problems % or *M* (*SD*)	*t* or χ^2^	*p*	Cohen’s* d*
Gender (boy)	53.5	70.9	13.44	< 0.001	–
Age T1	9.60 (1.27)	9.92 (1.33)	−2.59	0.010	0.12
Child self-esteem T1	3.38 (0.48)	3.08 (0.63)	5.50	< 0.001	−0.26
Child self-esteem T2	3.46 (0.45)	3.19 (0.57)	5.14	< 0.001	−0.25
Child self-esteem T3	3.53 (0.45)	3.23 (0.57)	5.14	< 0.001	−0.28
Maternal psychological control T1	2.13 (0.85)	2.63 (1.08)	−5.35	< 0.001	0.25
Maternal psychological control T2	1.87 (0.76)	2.38 (0.94)	−5.89	< 0.001	0.28
Maternal psychological control T3	1.88 (0.75)	2.43 (0.95)	−5.83	< 0.001	0.30
Paternal psychological control T1	2.17 (0.87)	2.55 (1.05)	−4.10	< 0.001	0.19
Paternal psychological control T2	1.94 (0.79)	2.34 (0.98)	−4.34	< 0.001	0.21
Paternal psychological control T3	1.84 (0.74)	2.38 (0.91)	−5.70	< 0.001	0.30

Table 2 shows the correlations between the study variables. In both groups, maternal psychological control showed small to moderate negative associations with children’s self-esteem at all time points. One exception to this general pattern was at T1, when this association was nonsignificant for children with oppositional defiant problems. In both groups, paternal psychological control showed small to moderate negative associations with children’s self-esteem at each time point.

**Table 2:** Correlations among the study variables

	1	2	3	4	5	6	7	8	9	10
1. Age	1	−0.08	−0.13	−0.21^⁎^	0.19^⁎⁎^	0.14^⁎^	0.20^⁎^	0.16^⁎^	0.10	0.05
2. Child self-esteem T1	−0.06	1	0.46^⁎⁎⁎^	0.26^⁎⁎^	−0.23^⁎⁎^	−0.12	−0.06	−0.23^⁎⁎^	−0.13	−0.12
3. Child self-esteem T2	0.03	0.40^⁎⁎⁎^	1	0.36^⁎⁎⁎^	−0.18^⁎^	−0.27^⁎⁎⁎^	−0.34^***^	−0.10	−0.21^⁎⁎^	−0.18^⁎^
4. Child self-esteem T3	0.06	0.38^⁎⁎⁎^	0.53^⁎⁎⁎^	1	−0.18^⁎^	−0.23^⁎⁎^	−0.34^⁎⁎⁎^	−0.13	−0.08	−0.26^⁎⁎^
5. Maternal psychological control T1	−0.04	−0.05	−0.20^⁎⁎^	−0.19^⁎^	1	0.39^⁎⁎⁎^	0.28^⁎⁎⁎^	0.61^⁎⁎⁎^	0.37^⁎⁎⁎^	0.27^⁎⁎^
6. Maternal psychological control T2	0.01	−0.09	−0.28^⁎⁎⁎^	−0.24^⁎⁎^	0.37^⁎⁎⁎^	1	0.40^⁎⁎⁎^	0.26^⁎⁎⁎^	0.62^⁎⁎⁎^	0.26^⁎⁎^
7. Maternal psychological control T3	0.03	−0.02	−0.08	−0.24^⁎⁎^	0.32^⁎⁎⁎^	0.41^⁎⁎⁎^	1	0.23^⁎⁎^	0.30^⁎⁎⁎^	0.60^⁎⁎⁎^
8. Paternal psychological control T1	−0.02	−0.20^⁎⁎^	−0.27^⁎⁎⁎^	−0.11	0.55^⁎⁎⁎^	0.25^⁎⁎⁎^	0.18^⁎^	1	0.46^⁎⁎⁎^	0.38^⁎⁎⁎^
9. Paternal psychological control T2	0.06	−0.09	−0.23^**^	−0.16*	0.21^⁎⁎^	0.66^⁎⁎⁎^	0.19^⁎^	0.30^⁎⁎⁎^	1	0.42^⁎⁎⁎^
10. Paternal psychological control T3	0.06	−0.04	−0.01	−0.19^⁎^	0.19^⁎^	0.25^⁎⁎^	0.69^⁎⁎⁎^	0.32^⁎⁎⁎^	0.29^⁎⁎⁎^	1

Note: Correlations for children with oppositional defiant problems are below the diagonal; those for children without oppositional defiant problems are above the diagonal.

^⁎^*p* < 0.05, ^⁎⁎^*p* < 0.01, ^⁎⁎⁎^*p* < 0.001

### Measurement invariance

We conducted two-group confirmatory factor analyses (CFA) to examine the metric and scalar invariance of the measures for children with and without oppositional defiant problems, and over time. Metric and scalar invariance are needed for making comparisons of associations and means, respectively (e.g., [70]). Following Little et al.’s [71] recommendations, we used parcels comprised of a set of items as indicators for latent constructs. Children’s self-esteem was represented by three parcels, each comprised of three items from the pertaining wave. Because parental psychological control was measured with five (rather than ten) items at T1, we cannot test its measurement invariance across three time points. Instead, we tested the measurement invariance of parents’ psychological control across T2 and T3. Parents’ psychological control was represented by three parcels at T2 and T3, each comprised of three to four items. The error terms for each parcel at each wave were allowed to correlate with the error terms for the corresponding parcels at the other waves [72]. Based on Chen’s [70] criteria for invariance (i.e., decrease in model fit of less than 0.01 for the CFI, and increase in model fit of less than 0.015 for the RMSEA) from the less to the more constrained model, the measures possessed metric and scalar invariance across groups (see Table 3). Partial scalar invariance was established for self-esteem and parental psychological control across measurement waves, with only one parcel that needed a freely estimated intercept at one time point. As long as at least two invariant indicators exist per measure, partial invariance allows meaningful comparisons using latent constructs [73]. We used observed mean values rather than the latent construct for ease of interpretation and comparability across studies.

**Table 3:** Measurement invariance models

Model	χ^2^	*df*	RMSEA	ΔRMSEA	CFI	ΔCFI	SRMR
Longitudinal invariance							
Self-esteem							
Configural	41.836	15	0.063		0.978		0.040
Metric	45.432	19	0.056	−0.007	0.979	0.001	0.044
Scalar	70.986	25	0.064	0.008	0.963	−0.016	0.057
Partial scalar	59.334	24	0.057	−0.007	0.971	−0.008	0.052
Paternal psychological control							
Configural	9.820	5	0.048		0.997		0.020
Metric	10.569	7	0.035	−0.013	0.998	0.001	0.021
Scalar	10.904	10	0.015	−0.020	0.999	0.001	0.022
Partial scalar	10.846	9	0.022	−0.013	0.999	0.000	0.022
Maternal psychological control							
Configural	5.286	5	0.012		1.000		0.012
Metric	8.632	7	0.023	0.011	0.999	−0.001	0.021
Scalar	10.660	10	0.012	−0.011	1.000	0.001	0.023
Between group invariance							
Self-esteem							
Configural	41.029	30	0.043		0.984		0.043
Metric	42.003	36	0.036	−0.007	0.991	0.007	0.045
Scalar	55.228	42	0.049	0.013	0.981	−0.010	0.052
Maternal psychological control							
Configural	17.288	10	0.059		0.994		0.030
Metric	25.662	14	0.063	0.004	0.991	−0.003	0.046
Scalar	30.061	18	0.056	−0.007	0.990	−0.001	0.049
Paternal psychological control							
Configural	15.969	10	0.053		0.996		0.027
Metric	21.502	14	0.051	−0.002	0.995	−0.001	0.040
Scalar	30.901	18	0.058	0.007	0.991	−0.004	0.046

### Associations between maternal psychological control and child self-esteem

**Fig. 1:**
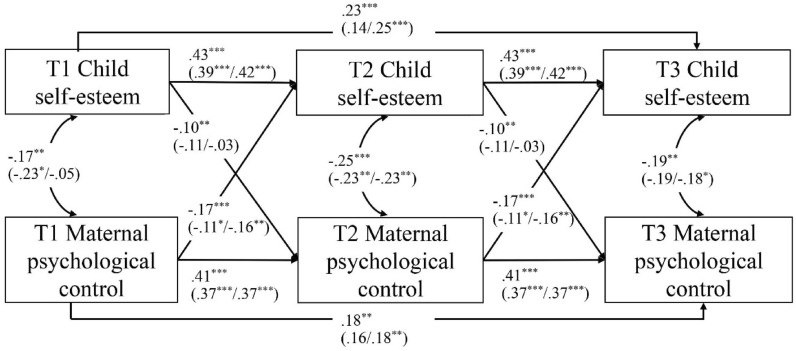
Bidirectional associations between maternal psychological control and child self-esteem across three time points (i.e., 2 years). Note: Standardized coefficients are presented for the total sample (and for children with and without oppositional defiant problems in brackets). We conducted additional analyses to explore potential child gender differences in the associations between maternal psychological control and children’s self-esteem (see Additional file 1: S2). We found no such child gender differences

The initial unconstrained CLPM showed an insufficient model fit, CFI = 0.94, TLI = 0.77, RMSEA = 0.12, SRMR = 0.05. To enhance the model fit, we added the autoregressive paths of T3 regressed on T1. Model fit improved significantly, CFI = 1.00, TLI = 0.97, RMSEA = 0.04, SRMR = 0.02. Next, we constrained autoregressive and cross-lagged paths to be equal from T1 to T2 and from T2 to T3. The time-constrained CLPM showed a similar model fit as the time-unconstrained model, CFI = 1.00, TLI = 1.01, RMSEA = 0.00, SRMR = 0.02; Δχ^2^ = 0.55, *p* = 0.969. Thus, these paths did not significantly differ across time. As such, we interpreted findings from the time-constrained CLPM (see Fig. 1).

Concurrent associations indicated that higher levels of psychological control were associated with lower levels of self-esteem at each measurement time, *r*s < −0.17, *p*s < 0.004. Autoregressive effects indicated that both maternal psychological control and children’s self-esteem showed considerable rank-order stability, βs ≥ 0.41, *p*s < 0.001. As hypothesized, both the cross-lagged maternal effect, β = −0.17, *p* < 0.001, and the cross-lagged child effect, β = −0.10, *p* = 0.007, were significant (see Table 4). Thus, higher levels of maternal psychological control predicted lower levels of child self-esteem one year later, and higher levels of child self-esteem predicted lower levels of maternal psychological control one year later. The post hoc power analysis (see Additional file 1: S4) indicated that our sample size (*n* = 447) was associated with a power of 89.40% to detect misspecifications of a CLPM corresponding to RMSEA = 0.08 on an alpha error of 0.05. With the available sample size, we had sufficient power (≥ 0.88) to detect all autoregressive effects (βs ≥ 0.41) and cross-lagged effects (βs ≤ −0.10).

**Table 4:** Model parameters of the time-constrained cross-lagged panel models

	Maternal model					Paternal model				
	*b*	*SE*	β	95% CI	*p*	*b*	*SE*	β	95% CI	*p*
Autoregressive paths										
Parental psychological control (T1 → T2)	0.36	0.04	0.41	[0.29, 0.43]	< 0.001	0.33	0.04	0.35	[0.25, 0.41]	< 0.001
Parental psychological control (T1 → T3)	0.16	0.05	0.18	[0.07, 0.26]	0.001	0.22	0.05	0.24	[0.12, 0.33]	< 0.001
Child self-esteem (T1 → T2)	0.40	0.04	0.43	[0.33, 0.47]	< 0.001	0.41	0.04	0.44	[0.34, 0.48]	< 0.001
Child self-esteem (T1 → T3)	0.21	0.05	0.23	[0.12, 0.31]	< 0.001	0.21	0.05	0.22	[0.11, 0.30]	< 0.001
Cross-lagged paths										
Parental effect (Parental psychological control T1 → Child self-esteem T2)	−0.09	0.02	−0.17	[−0.13, −0.05]	< 0.001	−0.07	0.02	−0.12	[−0.10, −0.03]	0.001
Child effect (Child self-esteem T1 → Parental psychological control T2)	−0.15	0.06	−0.10	[−0.27, −0.04]	0.007	−0.10	0.06	−0.06	[−0.21, 0.02]	0.104
Concurrent associations										
Parental psychological control T1 ↔ Child self-esteem T1	−0.10	0.03	−0.17	[−0.17, −0.03]	0.004	−0.14	0.03	−0.24	[−0.08, −0.14]	< 0.001
Parental psychological control T2 ↔ Child self-esteem T2	−0.09	0.02	−0.25	[−0.13, −0.05]	< 0.001	−0.08	0.02	−0.21	[−0.12, −0.04]	< 0.001
Parental psychological control T3 ↔ Child self-esteem T3	−0.06	0.02	−0.19	[−0.10, −0.02]	0.003	−0.08	0.02	−0.22	[−0.12, −0.04]	< 0.001

Note: In the time-constrained cross-lagged panel models, we constrained the autoregressive paths and cross-lagged paths to be identical from T1-T2 to T2-T3. Accordingly, we only report regression indices from T1-T2

Next, we conducted multigroup analyses to examine whether the cross-lagged effects between maternal psychological control and children’s self-esteem were different for children with and without oppositional defiant problems. The baseline model fitted the data well, CFI = 0.99, TLI = 0.98, RMSEA = 0.03, SRMR = 0.03. The fully constrained model fitted the data equally well, CFI = 1.00, TLI = 1.00, RMSEA = 0.00, SRMR = 0.04; Δχ^2^ = 3.07, *p* = 0.801. Compared to the baseline model, the “equal maternal effect” model, CFI = 0.99, TLI = 0.98, RMSEA = 0.03, SRMR = 0.03; Δχ^2^ = 0.49, *p* = 0.484, and the “equal child effect” model, CFI = 0.99, TLI = 0.98, RMSEA = 0.04, SRMR = 0.03; Δχ^2^ = 1.34, *p* = 0.248, fitted the data equally well (see Table 4). Thus, the results indicated that both the maternal effect and the child effect did not differ between groups.

We also adopted RI-CLPM to disentangle between-person from within-person effects [64]. We found a strong negative between-person association between self-esteem and maternal psychological control (*r* = −0.53, *p* < 0.001), indicating that children with lower self-esteem generally reported more maternal psychological control relative to other children across three measurement waves. However, we found no significant within-person level associations between self-esteem and parental psychological control, both concurrently and longitudinally. That is, children who scored higher or lower than their average level of self-esteem did not report increases or decreases in maternal psychological control score at the same time or later (see Additional file 1: S3). Based on the insights provided from the CLPM and RI-CLPM, we found that the associations between maternal psychological control and children’s self-esteem primarily manifest as between-person effects.

We were not able to make a group comparison between children with and without oppositional defiant problems, because the RI-CLPM did not converge for children with oppositional defiant problems. Previous studies have suggested that RI‐CLPM is computationally more complex and therefore has lower power to detect effects compared to CLPM [74]. Indeed, post hoc power analysis (see Additional file 1: S4) indicated that our sample size (*n* = 447) is associated with a power of 39.35% to detect misspecifications of a RI-CLPM corresponding to RMSEA = 0.08 for an alpha error of 0.05. With the available sample size, we had insufficient power (≤ 0.30) to detect all autoregressive effects (βs ≥ 0.01) and cross-lagged effect in RI-CLPM (βs ≤ −0.01). Even with a sample of *n* = 10,000, there would not have been enough power to detect effects of self-esteem at T1 on self-esteem at T2, and of maternal psychological control at T1 on self-esteem at T2.

We conducted one additional CLPM in which oppositional defiant problems were treated as a continuous variable (rather than as a grouping variable). This allowed us to examine the associations between children’s oppositional defiant problems, self-esteem and maternal psychological control over time (see  Additional file 1: S5). We found that maternal psychological control and children’s oppositional defiant problems negatively predicted child self-esteem one year later, while children’s oppositional defiant problems positively predicted maternal psychological control one year later. These findings illustrate how consideration of children’s oppositional defiant problems can add to our understanding of the associations between parental psychological control and child self-esteem.

### Associations between paternal psychological control and child self-esteem

**Fig. 2:**
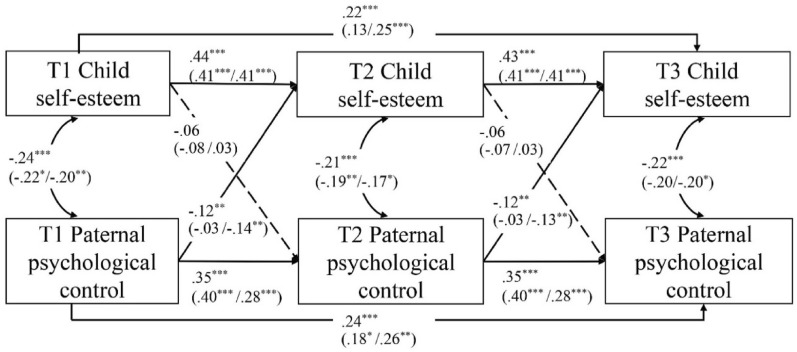
Bidirectional associations between paternal psychological control and child self-esteem across three timepoints (i.e., 2 years). Note: Standardized coefficients are presented for the total sample (and for children with and without oppositional defiant problems in brackets). The dotted lines indicate non-significant associations. We conducted additional analyses to explore potential child gender differences in the associations between paternal psychological control and children’s self-esteem (see Additional file 1: S2). We found no such child gender differences

The initial unconstrained CLPM showed an insufficient model fit, CFI = 0.92, TLI = 0.68, RMSEA = 0.14, SRMR = 0.06. To enhance the model fit, we added the autoregressive paths of T3 regressed on T1. Model fit improved significantly, CFI = 1.00, TLI = 1.03, RMSEA = 0.00, SRMR = 0.01. Next, we constrained autoregressive and cross-lagged paths to be equal from T1 to T2 and from T2 to T3. The time-constrained CLPM showed a similar model fit as the time-unconstrained model, CFI = 1.00, TLI = 1.03, RMSEA = 0.00, SRMR = 0.01; Δχ^2^ = 1.61, *p* = 0.808. Thus, these paths did not significantly differ across time. We interpreted findings from the time-constrained CLPM (see Fig. 2).

Concurrent associations indicated that higher levels of paternal psychological control were associated with lower levels of self-esteem at each measurement time, *rs* < −0.21, *p*s < 0.001. Autoregressive effects indicated that both paternal psychological control and children’s self-esteem showed considerable rank-order stability, βs ≥ 0.35, *p*s < 0.001. As hypothesized, the cross-lagged paternal effect was significant, β = −0.12, *p* = 0.001. However, this was not the case for the cross-lagged child effect, β = −0.06, *p* = 0.104. Thus, higher levels of paternal psychological control predicted lower levels of child self-esteem one year later, but higher levels of child self-esteem did not predict lower levels of paternal psychological control one year later (see Table 4). The post hoc power analysis (see Additional file 1: S4) indicated that our sample size (*n* = 447) was associated with a power of 89.40% to detect misspecifications of a CLPM corresponding to RMSEA = 0.08 for an alpha error of 0.05. With the available sample size, we had sufficient power (≥ 0.97) to detect all autoregressive effects and the effect of paternal psychological control at T1 on self-esteem at T2, but not to detect the effect of self-esteem at T1 on paternal psychological control at T2 (power = 0.48). With a sample of *n* = 1000, power would have been sufficient (≥ 0.82) to detect all autoregressive (βs ≥ 0.35) and cross-lagged effects (βs ≤ −0.06).

Next, we conducted multigroup analyses to examine whether the cross-lagged effects between paternal psychological control and child self-esteem were different for children with and without oppositional defiant problems. The baseline model fitted the data well, CFI = 1.00, TLI = 1.02, RMSEA = 0.00, SRMR = 0.03. The fully constrained model fitted the data equally well, CFI = 1.00, TLI = 1.00, RMSEA = 0.00, SRMR = 0.05; Δχ^2^ = 7.69, *p* = 0.261, indicating that cross-lagged effects were similar in both groups of children. Compared to the baseline model, the “equal paternal effect” model, CFI = 1.00, TLI = 1.01, RMSEA = 0.00, SRMR = 0.03; Δχ^2^ = 2.32, *p* = 0.128, and the “equal child effect” model, CFI = 1.00, TLI = 1.01, RMSEA = 0.00, SRMR = 0.04; Δχ^2^ = 2.09, *p* = 0.148, fitted the data equally well. Thus, the findings indicated that both the paternal effect and the child effect did not differ across groups (see Table 5).

**Table 5:** Model comparison of reciprocal associations for children with and without oppositional defiant problems

	Children without oppositional defiant problems					Children with oppositional defiant problems					Group difference (Δχ^2^)
	*b*	*SE*	*β*	95% CI	*p*	*b*	*SE*	*β*	95% CI	*p*	
Maternal model											
Maternal effect	−0.06	0.03	−0.11	[−0.12, −0.00]	0.041	−0.09	0.03	−0.16	[−0.14, −0.03]	0.001	0.49
Child effect	−0.18	0.10	−0.11	[−0.37, 0.01]	0.068	−0.04	0.07	−0.03	[−0.19, 0.10]	0.560	1.34
Paternal model											
Paternal effect	−0.02	0.03	−0.03	[−0.07, 0.04]	0.551	−0.07	0.03	−0.14	[−0.13, −0.02]	0.005	2.32
Child effect	−0.12	0.10	−0.08	[−0.33, 0.08]	0.238	0.05	0.08	0.03	[−0.10, 0.20]	0.526	2.09

We also conducted RI-CLPM [64]. We found a strong negative between-person association between self-esteem and paternal psychological control (*r* = −0.46, *p* < 0.001), indicating that children with relatively lower self-esteem tended to report relatively more paternal psychological control across three measurement waves. We found no significant within-person level associations between self-esteem and paternal psychological control, both concurrently and longitudinally. That is, children who scored higher or lower than their expected self-esteem score did not report increases or decreases in paternal psychological control score at the same time, or later (see Additional file 1: S3). Based on the insights provided from the CLPM and RI-CLPM, we found that the associations between paternal psychological control and children’s self-esteem primarily manifest as between-person effects.

We were not able to make a group comparison between children with and without oppositional defiant problems, because the RI-CLPM did not converge in the group of children with oppositional defiant problems. The post hoc power analysis (see Additional file 1: S4) indicated that our sample size (*n* = 447) was associated with a power of 84.66% to detect misspecifications of a RI-CLPM corresponding to RMSEA = 0.08 for an alpha error of 0.05. With the available sample size, we had insufficient power (≤ 0.18) to detect all autoregressive effects (βs ≥ 0.02) and cross-lagged effects (βs ≤ −0.03) in RI-CLPM. Even with a sample of *n* = 10,000, there would not have been enough power to detect effects of paternal psychological control at T1 on paternal psychological control at T2.

We also examined the associations between children’s ODD symptoms, self-esteem and paternal psychological control over time (see Additional file 1: S5). We found that paternal psychological control and children’s oppositional defiant problems negatively predicted child self-esteem one year later. Children’s oppositional defiant problems and paternal psychological control showed a mutually reinforcing pattern, such that children’s oppositional defiant problems positively predicted paternal psychological control one year later and vice versa.

## Discussion

The present longitudinal study contributes understanding of the self-esteem development of children with (vs. without) oppositional defiant problems, and it does so in the important but relatively understudied cultural context of China. Specifically, we tested longitudinal and bi-directional associations between children’s self-esteem and their parents’ psychological control. Compared to their peers without oppositional defiant problems, children with oppositional defiant problems reported lower levels of self-esteem, and higher levels of both maternal and paternal psychological control. The pattern of associations between children’s self-esteem and parental psychological control, however, was similar for children with and without oppositional defiant problems. Results for the total sample revealed bi-directional associations between maternal psychological control and children’s self-esteem (i.e., parent- and child-effects). Children who perceived higher levels of psychological control from their mothers were likely to exhibit lower levels of self-esteem over time, and conversely, children who exhibited lowed levels of self-esteem were likely to perceive more maternal psychological control over time. Results for the total sample revealed a uni-directional association between paternal psychological control and children’s self-esteem (i.e., parent-effect only). Children who perceived higher levels of psychological control from their fathers were likely to exhibit lower levels of self-esteem over time. Together, these results suggest that parents’ psychologically controlling behaviors compromise their children’s self-esteem, which, in turn, may lead children to perceive even more psychologically controlling behaviors from their mothers over time.

### Parental psychological control predicting decreased child self-esteem

We extended prior research (e.g., [27, 28]) by demonstrating that negative effects of psychological control on children’s self-esteem can be found for both mothers and fathers. We did so in the cultural context of China, where psychological control is more normative and accepted as compared to Western cultures [75, 76]. Our findings resonate with the perspective that parental psychological control may compromise children’s self-esteem by thwarting the universal need for autonomy [36]—thus, the self-esteem eroding effect of parental psychological control extends beyond parent roles and cultural borders.

Different from what we hypothesized, the self-esteem of children with (vs. without) oppositional defiant problems was not more susceptible to adverse effects of parental psychological control. A possible explanation is that children’s oppositional defiant behaviors may serve a protective role, helping children to ward off negative self-views [13]. Psychological reactance theory supports this view, suggesting that oppositional defiant behaviors may represent attempts to reclaim threatened autonomy or agency [77–79]. While such oppositionality or defiance may be counterproductive in negotiating autonomy with parents [80, 81], these behaviors might enable children to avoid or cope with autonomy thwarting experiences (e.g., caused by psychological control), and prevent them from experiencing particularly strong decreases in self-esteem (that is, stronger decreases than are experienced by children without oppositional defiant problems). Another possibility is that children without oppositional defiant problems may experience other psychological problems (e.g., symptoms of anxiety, depression) that potentially obscure the anticipated differences between groups. Future research could benefit from a more encompassing approach, examining the influence of various mental health comorbidities, along with oppositional defiant problems, on the association between parental psychological control and child self-esteem.

### Low child self-esteem predicting parental psychological control

Building on transactional theory [10], our study is the first to demonstrate that children’s level of self-esteem predicts perceived parental psychological control practices. That is, children’s lower levels of self-esteem predicted increased psychological control over time from mothers (which, in turn, further decreased children’s self-esteem), but not fathers. These differential effects are in line with prior evidence that mothers, but not fathers, tend to employ psychologically controlling practices when they perceive their children as emotionally vulnerable or socially less competent [38]. We demonstrated this effect in China, a cultural context where family responsibilities tend to be traditional. For example, Chinese mothers are more often tasked than fathers with daily childcare and addressing emotional needs of their children [50–52]. Relatedly, it is possible that, in our study, fathers were less aware of their children’s level of self-esteem in the first place, for example because they tend to spend less time with their children [53, 82].

While children with oppositional defiant problems reported perceiving more psychological control than their counterparts without oppositional defiant problems, we found—different from what we hypothesized—that the predictive effects of self-esteem on parental psychological control were actually similar for both groups of children. This is somewhat surprising from an accumulated risk perspective, which would suggest that parents will be especially likely to resort to psychological control when their children suffer from both low self-esteem and oppositional defiant problems. We speculate that children’s overt oppositional defiance may be more conspicuous and pressing for their parents, possibly overshadowing more subtle vulnerabilities such as low self-esteem. We found support for this possibility in our supplementary analyses (Additional file 1: S5), which showed that children’s oppositional defiant problems, but not their self-esteem, predict parental psychological control. Parents may be inclined to engage in psychological control to try to make sure their children with oppositional defiant problems live up to societal norms and expectations. This hypothesis presents an interesting direction for future research.

### Limitations and future directions

Our study has a number of limitations. First, while we implemented a rigorous selection procedure to identify children with oppositional defiant problems, these children were not clinically diagnosed. Instead, two clinical psychologists based their selection on teachers’ reports, which may have been skewed towards children whose ODD symptoms manifest predominantly in a school environment. We cannot rule out the possibility that we would have found more or other differences between children with and without oppositional defiant problems (e.g., in terms of the associations between self-esteem and parental psychological control) if we had sampled children with clinically diagnosed ODD. Future research could test this possibility.

Another limitation is that we relied solely on children’s perceptions of parental psychological control. While children’s perceptions of how their parents act toward them are central to their self-esteem development [16], our exclusive reliance on children’s self-reports may have introduced some confounds. For instance, children with high self-esteem might view their parents relatively favorably (i.e., ‘halo effect’), which may be reflected in their reports of their parents’ psychological control. Additionally, children’s subjective interpretations of their parents’ behaviors typically are only partially overlapping with those of their parents themselves, or of other observers [83]. Future research with multi-informant measures (e.g., parental reports, observations) of parental psychological control will provide a more comprehensive understanding of the association between parental psychological control and children’s self-esteem.

We theorized that children’s (thwarted) need for autonomy might be the psychological driver that explains why parental psychological control predicts decreases in children’s self-esteem [36]. However, we did not empirically test this assumption. Future research could scrutinize the psychological mechanism that accounts for why or how psychologically controlling parenting affects children’s self-development.

Finally, we acknowledge the cultural specificity of our findings. Our study contributes to the literature by examining the self-development of children growing up in the non-Western and relatively understudied context of China. That said, we encourage cross-cultural follow-up research to build well-rounded understanding of potential cultural variation in the dynamics between psychologically controlling parenting behaviors and children’s self-esteem.

## Conclusion and implications

Our findings showed both differences and similarities in Chinese children with and without oppositional defiant problems, in terms of levels of and associations between self-esteem and parental psychological control. Children with oppositional defiant problems not only reported lower self-esteem, but also perceived more psychological control from their parents. This insight informs professionals (e.g., educators, clinicians), and points to the necessity for interventions that address both the behavioral symptoms and the underlying mechanisms affecting self-esteem. However, we found similar associations between self-esteem and parental psychological control in children with and without oppositional defiant problems, which suggests an underlying mechanism that transcends the binary categorization of mental health problems. Specifically, parental psychological control compromises children’s self-esteem regardless of children’s oppositional defiant problems, which highlights the important role parents play in shaping their children’s self-esteem. Parenting interventions could aim to increase parental awareness of the adverse effects of psychological control. Moreover, our findings illustrate that children with low self-esteem may perceive increased psychological control from their mothers (but not fathers), which offers valuable insight to within-family dynamics, and underscores the importance for parenting intervention to break negative cycles of parent–child influence. Psychological education programs could teach parents strategies for positive behavior reinforcement, without resorting to psychological control. We hope our study will spur future research to better understand parenting strategies that shape healthy self-development in children with and without behavioral problems, in China and across the globe.

### Supplementary Information

**Additional file 1:** **S1.** Development of self-esteem and group comparison. **S2.** Gender differences in the associations between parental psychological control and child self-esteem. **S3.** Exploratory analysis of random intercept cross lagged panel model. **S4.** Power analysis. **S5.** Associations between parental psychological control, child self-esteem and ODD symptoms.

## Data Availability

The dataset for the analyses are available on the OSF page for our study (https://osf.io/5wqrd/).

## Footnotes

1 Due to planned missingness at T1, we generated the T1 maternal and paternal psychological control scores based on five rather than ten items. The 5-item score correlated very strongly with the 10-item score at T2 and T3, with *r*s ranging from 0.94 to 0.96, and from 0.94 to 0.95, for mothers’ and fathers’ psychological control, respectively).
