# Supplementary material for: Parental psychological control and children’s self-esteem: A longitudinal investigation in children with and without oppositional defiant problems
Source: Child Adolesc Psychiatry Ment Health. 2024 Apr 29;18:50. doi: 10.1186/s13034-024-00740-0 (PMC11059723; doi:10.1186/s13034-024-00740-0)
Supplement: Supplementary file 1 — Additional file 1: S1. Development of self-esteem and group comparison. S2. Gender differences in the associations between parental psychological control and child self-esteem. S3. Exploratory analysis of random intercept cross lagged panel model. S4. Power analysis. S5. Associations between parental psychological control, child self-esteem and ODD symptoms. [file 13034_2024_740_MOESM1_ESM.docx]

**Supplementary Material**

[S1 Development of Self-Esteem and Group Comparison 2](#_Toc160531626)

[S2 Gender Differences in the Associations Between Parental Psychological Control and Child Self-Esteem 4](#_Toc160531627)

[S3 Exploratory Analysis of Random Intercept Cross Lagged Panel Model 6](#_Toc160531628)

[S4 Power Analysis 9](#_Toc160531629)

[S5 Associations Between Parental Psychological Control, Child Self-Esteem and ODD Symptoms 11](#_Toc160531630)

**S1 Development of Self-Esteem and Group Comparison**

We conducted latent growth curve models (LGM; Duncan et al., 2013) to estimate the initial level (intercept) and change (linear slope) in children’s self-esteem across three measurement waves (with one-year intervals). As there were only three measurement waves, we restricted our model to estimate linear slopes only. Given the substantial age heterogeneity at each wave, we used the TSCORES function in Mplus to scale the intercept and linear slope factors on children’s age estimated as individually varying time indicators (Mehta & West, 2000). We define the intercept of the growth factor at the lowest possible age in the sample (i.e., age 8) by subtracting 8 from all T-scores (i.e., ageT1 - 8, ageT2 - 8, and ageT3 - 8). We built the latent growth model for the self-esteem of children with and without oppositional behavioral problems (ODD) in a multi-group LGM. We tested group differences in intercepts and slopes using Wald tests. We conducted the analyses in Mplus 8.0 (Muthén & Muthén, 2017).

**Results**

Parameter estimates of intercept and slope of children’s self-esteem for both groups are depicted in Table S1. Because our model included individually varying times of observation, resulting in a multilevel structure, it was not possible to obtain standardized coefficients for the prediction models. Accordingly, we report unstandardized coefficients.

Group comparisons indicated that children with ODD reported lower initial levels of self-esteem, intercept = 3.05, *p* < .001, compared to children without ODD, intercept = 3.43, *p* < .001, Δχ^2^ = 27.01, *p* < .001. Children with ODD showed an increase in self-esteem over time, linear slope = 0.04, *p* = .010, whereas the self-esteem level of children without ODD was stable over time, linear slope = 0.01, *p* = .631. Yet, the linear slopes did not differ significantly between the two groups, Δχ^2^ = 2.43, *p* = .119. The correlations between intercept and slope of self-esteem were not significant in both children with ODD, *r* = -.01, *p* = .874, and without ODD, *r* = -.01, *p* = .807. The strength of the correlations between intercept and slope of self-esteem did not differ between the two groups, Δχ^2^ = 0.07, *p* = .791. Thus, compared to children without behavioral problems, children with ODD report lower levels of self-esteem. Their self-esteem increases across the study period, but their slope of self-esteem change is not significantly different from the one observed in children without ODD.

**Table S1**

*Self-Esteem Development in Children With and Without Oppositional Defiant Problems*

|  | Estimate (*SE*) | | | Group difference  (Wald test) |
| --- | --- | --- | --- | --- |
| Estimate | Children without oppositional defiant problems |  | Children with oppositional defiant problems |  |
| **Children’s self esteem** |  |  |  |  |
| **Intercept** |  |  |  |  |
| Mean | 3.43 (0.04)^⁎⁎⁎^ |  | 3.05 (0.06)^⁎⁎⁎^ | 27.01^⁎⁎⁎^ |
| Variance | 0.05 (0.05) |  | 0.18 (0.09)^⁎^ |  |
| **Slope** |  |  |  |  |
| Mean | 0.01 (0.02) |  | 0.04 (0.02)^⁎^ | 2.43 |
| Variance | 0.002 (0.007)^a^ |  | 0.001 (0.01) ^a^ |  |
| **Correlation between intercept and slope** | -0.01 (0.02) |  | -0.01 (0.03) | 0.07 |

*Note.*

^⁎^*p* < .05, ^⁎⁎^*p* < .01, ^⁎⁎⁎^*p* < .001.

a. Estimates are presented in three decimals as they represent very small effects.

**S2 Gender Differences in the Associations Between Parental Psychological Control and Child Self-Esteem**

We conducted multiple group models to examine whether the cross-lagged effects between paternal psychological control and child self-esteem were different for boys and girls. The baseline model, CFI = 0.98, TLI = 0.94, RMSEA = .06, SRMR = .04, and the fully constrained model constraining all cross-lagged effects to be identical across groups fitted the data equally well, CFI = 0.97, TLI = 0.96, RMSEA = .05, SRMR = .05; Δχ²= 6.67, *p* = .352. Compared to the baseline model, the “equal maternal effect” model, CFI = 0.97, TLI = 0.94, RMSEA = .06, SRMR = .05; Δχ²= 1.86, *p* =.173, and the “equal child effect” model, CFI = 0.98, TLI = 0.95, RMSEA = .06, SRMR = .04; Δχ²= 0.002, *p* =.964, fitted the data equally well (see Table S2). Thus, the results indicated that both the maternal effect and the child effect did not differ across groups.

Next, we conducted multiple group models to examine whether the cross-lagged effects between paternal psychological control and child self-esteem were for boys and girls. The baseline model, CFI = 0.99, TLI = 0.98, RMSEA = .03, SRMR = .04, and the fully constrained model constraining all cross-lagged effects to be identical across groups fitted the data equally well, CFI = 1.00, TLI = 1.01, RMSEA = .00, SRMR = .04; Δχ²= 3.07, *p* = .800. Compared to the baseline model, the “equal paternal effect” model, CFI = 1.00, TLI = 0.99, RMSEA = .03, SRMR = .04; Δχ²= 0.01, *p* =.931, and the “equal child effect” model, CFI = 0.99, TLI = 0.99, RMSEA = .03, SRMR = .04; Δχ²= 0.26, *p* =.613, fitted the data equally well (see Table S3). Thus, the results indicated that both the paternal effect and the child effect did not differ across groups.

**Table S2**

*Model Comparison of Reciprocal Associations Boys and Girls*

|  |  | Boys | | | |  |  | Girls | | | | Group difference (Δχ^2^) |
| --- | --- | --- | --- | --- | --- | --- | --- | --- | --- | --- | --- | --- |
|  | *b* | *SE* | β | 95% CI | *p* | | *b* | *SE* | β | 95% CI | *p* |  |
| **Maternal model** |  |  |  |  |  | |  |  |  |  |  |  |
| Maternal effect | -0.07 | .02 | -0.13 | [-0.12, -0.02] | .004 | | -0.13 | .04 | -0.25 | [-0.20, -0.06] | <.001 | 1.86 |
| Child effect | -0.13 | .07 | -0.09 | [-0.28, 0.01] | .068 | | -0.14 | .10 | -0.08 | [-0.33, 0.05] | .156 | 0.002 |
| **Paternal model** |  |  |  |  |  | |  |  |  |  |  |  |
| Paternal effect | -0.05 | .03 | -0.09 | [-0.10, -0.001] | .044 | | -0.05 | .03 | -0.09 | [-0.10, 0.01] | .079 | 0.01 |
| Child effect | -0.05 | .08 | -0.03 | [-0.21, 0.12] | .585 | | 0.02 | .11 | 0.01 | [-0.19, 0.24] | .835 | 0.26 |

**S3 Exploratory Analysis of Random Intercept Cross Lagged Panel Model**

**Maternal Model**

The model fit of the unconstrained RI-CLPM was acceptable, CFI = 0.99, TLI = 0.89, RMSEA = 0.08, SRMR = 0.02. We constrained autoregressive and cross-lagged paths to be equal from T1 to T2 and from T2 to T3. The time-constrained model showed significant better model fit than the time-unconstrained model, CFI = 0.99, TLI = 0.97, RMSEA =.04, SRMR = .05. Thus, these paths significantly differ across time. We interpreted findings from the time-unconstrained model (see Figure S1).

The between-person association between self-esteem and maternal psychological control was strong and negative, indicating that children with lower self-esteem across the measurement waves reported more maternal psychological control across measurement waves than individuals with low self-esteem. On the within-person level, we did not find significant concurrent associations between self-esteem and maternal psychological control at T1 and T2, but we found that children who scored higher or lower than their average self-esteem score tended to score higher or lower than their average maternal psychological control score on T3. We found no any within-person cross-lagged effects, indicating that children who scored higher or lower than their average self-esteem score did not report higher or lower than their average maternal psychological control score at the next assessment, and vice versa. There were no significant carry-over stability effects of self-esteem and maternal psychological control. Within-person deviations from the average self-esteem and maternal psychological control do not predict deviations from the average self-esteem and maternal psychological control at the next time point.


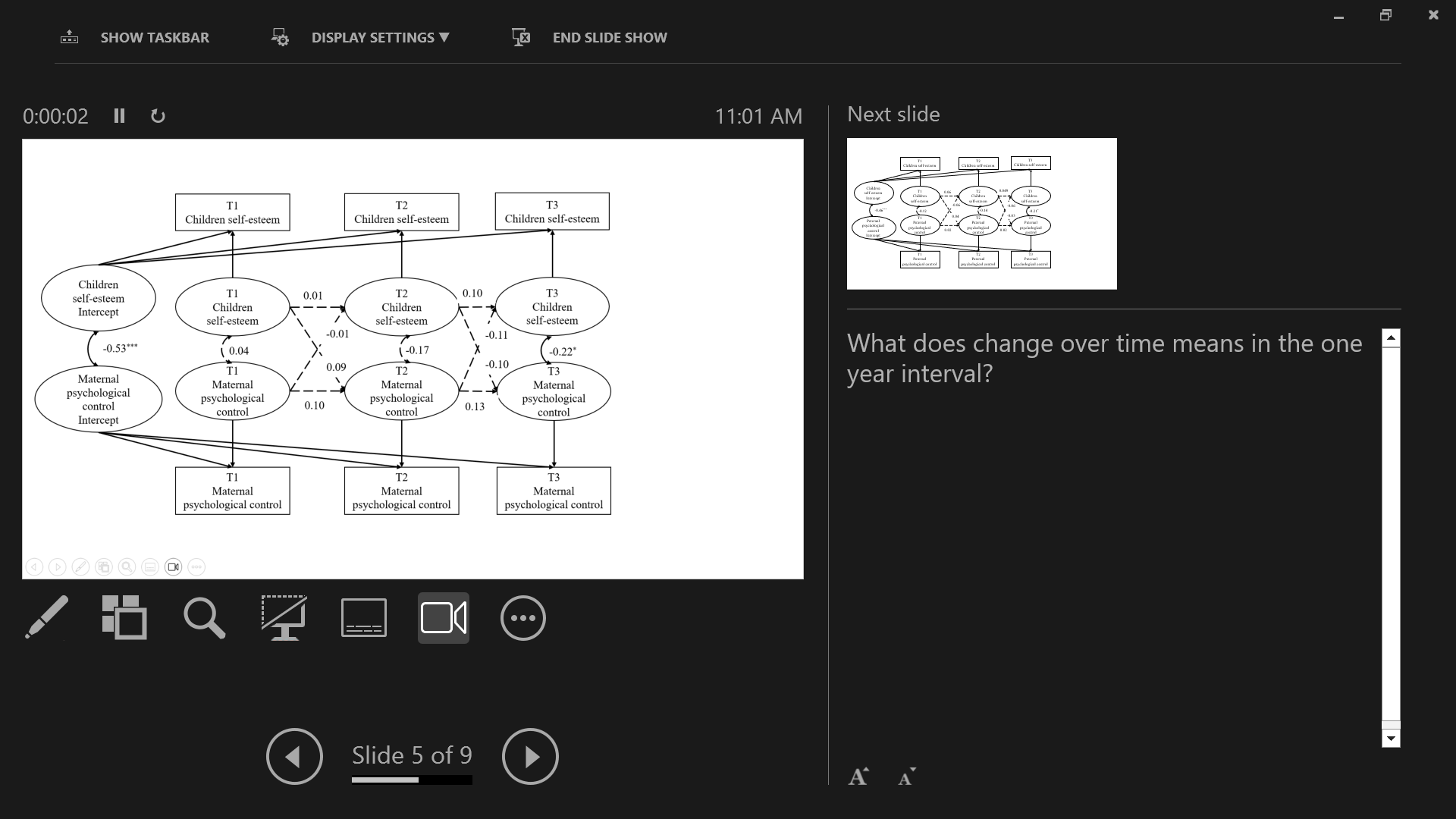


*Figure S1.* RI-CLPM exploring bidirectional associations between maternal psychological control and child self-esteem across three timepoints (i.e., two years).

**Paternal Model**

The model fit of the unconstrained RI-CLPM was good, CFI = 1.00, TLI = 1.04, RMSEA = 0.00, SRMR = 0.00. We constrained autoregressive and cross-lagged paths to be equal from T1 to T2 and from T2 to T3. The time-constrained model showed a similar model fit as the time-unconstrained model, CFI = 1.00, TLI = 1.03, RMSEA =.00, SRMR = .03; Δχ²= 1.61, *p* =.808. Thus, these paths did not significantly differ across time. We interpreted findings from the time-constrained model (see Figure S2).

The between-person association between self-esteem and maternal psychological control was strong and negative, indicating that individuals with lower self-esteem across the measurement waves reported higher paternal psychological control across measurement waves than individuals with low self-esteem. On the within-person level, we did not find significant concurrent associations between self-esteem and paternal psychological control at T1 and T2, but we found that children who scored higher or lower than their average self-esteem score tended to score higher or lower than their average paternal psychological control score on T3. We found no any within-person cross-lagged effects, indicating that children who scored higher or lower than their average self-esteem score did not report higher or lower than their average paternal psychological control score at the next assessment, and vice versa. There were no significant carry-over stability effects of self-esteem and paternal psychological control. Within-person deviations from the average self-esteem and paternal psychological control do not predict deviations from the average self-esteem and paternal psychological control at the next time *
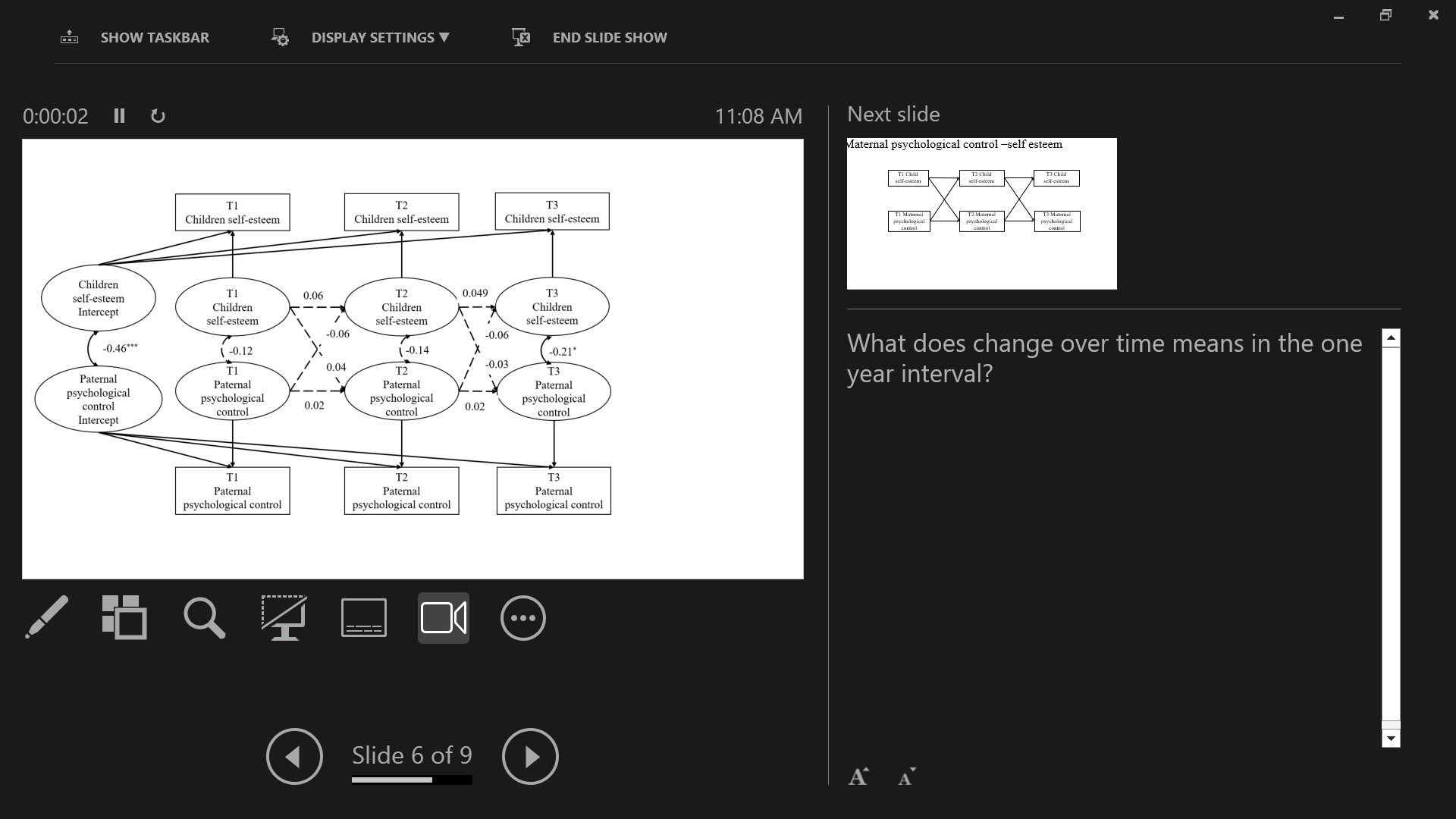
*point.

*Figure S2.* RI-CLPM exploring bidirectional associations between maternal psychological control and child self-esteem across three timepoints (i.e., two years).

**S4 Power Analysis**

We calculated power for each estimated pathway in our models by means of a Monte Carlo simulation in Mplus 8.0 (Muthén & Muthén, 2017). To estimate the models, effect sizes for all the paths in the models had to be specified. We therefore used the estimates provided by our CLPM and RI-CLPM results, disregarding significance level, as input for the power analysis of both the RI-CLPM and the CLPM. To do this, we first re-estimated our models in Mplus and saved the starting values. Then, we ran a Monte Carlo simulation for each model, using the starting values as input. We simulated the achieved power with the actual sample size as well as with increasingly large samples up to 10,000, to test the required sample size for sufficient power (≥ .80) for each estimated pathway. The results of the power analyses are presented in Table S3. We discuss the results from the power analyses in the respective paragraphs of the Results section.

**Table S3**

*Power Analyses RI-CLPM and CLPM*

|  | **T1-T2** | **T1-T2** | **T1-T2** | **T1-T2** | **T2-T3** | **T2-T3** | **T2-T3** | **T2-T3** | **Intercept** |
| --- | --- | --- | --- | --- | --- | --- | --- | --- | --- |
|  | **SE- SE** | **PC-PC** | **SE- PC** | **PC- SE** | **SE- SE** | **PC-PC** | **SE- PC** | **PC- SE** | **PC -SE** |
| Maternal CLPM 447 | 1 | 1 | 0.877 | 1 | 1 | 1 | 0.877 | 1 | - |
| 1000 | 1 | 1 | 0.996 | 1 | 1 | 1 | 0.996 | 1 | - |
| 2000 | 1 | 1 | 1 | 1 | 1 | 1 | 1 | 1 | - |
| 10000 | 1 | 1 | 1 | 1 | 1 | 1 | 1 | 1 | - |
| Maternal Ri-CLPM 447 | 0.056 | 0.198 | 0.171 | 0.060 | 0.208 | 0.299 | 0.250 | 0.255 | 0.991 |
| 1000 | 0.052 | 0.378 | 0.329 | 0.061 | 0.360 | 0.530 | 0.487 | 0.498 | 1 |
| 2000 | 0.052 | 0.640 | 0.582 | 0.059 | 0.582 | 0.815 | 0.775 | 0.780 | 1 |
| 10000 | 0.054 | 0.999 | 0.999 | 0.109 | 0.996 | 1 | 1 | 1 | 1 |
| Paternal CLPM 447 | 1 | 1 | 0.482 | 0.967 | 1 | 1 | 0.482 | 0.967 | - |
| 1000 | 1 | 1 | 0.817 | 1 | 1 | 1 | 0.817 | 1 | - |
| 2000 | 1 | 1 | 1 | 1 | 1 | 1 | 1 | 1 | - |
| 10000 | 1 | 1 | 1 | 1 | 1 | 1 | 1 | 1 | - |
| Paternal Ri-CLPM 447 | 0.109 | 0.062 | 0.098 | 0.177 | 0.109 | 0.062 | 0.098 | 0.177 | 0.994 |
| 1000 | 0.174 | 0.065 | 0.143 | 0.308 | 0.174 | 0.065 | 0.143 | 0.308 | 1 |
| 2000 | 0.300 | 0.078 | 0.227 | 0.542 | 0.300 | 0.078 | 0.227 | 0.542 | 1 |
| 10000 | 0.901 | 0.181 | 0.766 | 0.997 | 0.901 | 0.181 | 0.766 | 0.997 | 1 |

Note. SE represent self-esteem, PC represent parental psychological control.

**S5 Associations Between Parental Psychological Control, Child Self-Esteem and ODD Symptoms**

To test associations between parental psychological control, children’s self-esteem and ODD symptoms, we conducted three-wave Cross-Lagged Panel Models (CLPM). In these models, concurrent associations (i.e., the correlation between maternal/paternal psychological control, children’s self-esteem and ODD symptoms) were included at each measurement time. Autoregressive paths (i.e., the predictive effect of a variable on itself) were included for both maternal psychological control, children’s self-esteem and ODD symptoms. Cross-lagged paths (i.e., the predictive effect of one variable on another variable) were included as well. For the sake of parsimony, we constrained autoregressive and cross-lagged paths to be equal from T1 to T2 and from T2 to T3. We used Chi-square difference testing to examine whether the model fit differed significantly between the unconstrained and the constrained model. If the constrained model did not fit significantly worse, we interpreted findings from the constrained model.

**Maternal Model**

The initial unconstrained cross-lagged panel model showed good model fit, CFI = 0.99, TLI = 0.93, RMSEA = .06, SRMR = .02^[[1]](#footnote-1)^. Next, we constrained autoregressive and cross-lagged paths to be equal from T1 to T2 and from T2 to T3. The time-constrained model showed a similar model fit as the time-unconstrained model, CFI = 0.99, TLI = 0.98, RMSEA =.03, SRMR = .03; Δχ²= 6.95, *p* =.642. Thus, these paths did not significantly differ across time. As such, we interpreted findings from the time-constrained model (see Figure S3).

Concurrent associations indicated that higher levels of maternal psychological control were associated with lower levels of self-esteem at each measurement time, *r*s < -.17, *p*s < .004. Higher levels of ODD symptoms were associated with lower levels of self-esteem at T1 and T2, *r*s < -.18, *p*s < .001. Higher levels of ODD symptoms were associated with higher levels of maternal psychological control at T1, *r* = .26, *p* < .001. Autoregressive effects indicated that both maternal psychological control, children’s self-esteem and ODD symptoms showed considerable rank-order stability, βs > 0.38, *p*s < .001. Cross-lagged effects indicated that, higher levels of maternal psychological control predicted lower levels of child self-esteem one year later, β = -0.14, *p* < .001, higher levels of child ODD symptoms predicted lower levels of child self-esteem one year later, β = -0.10, *p* = .008, and higher levels of maternal psychological control one year
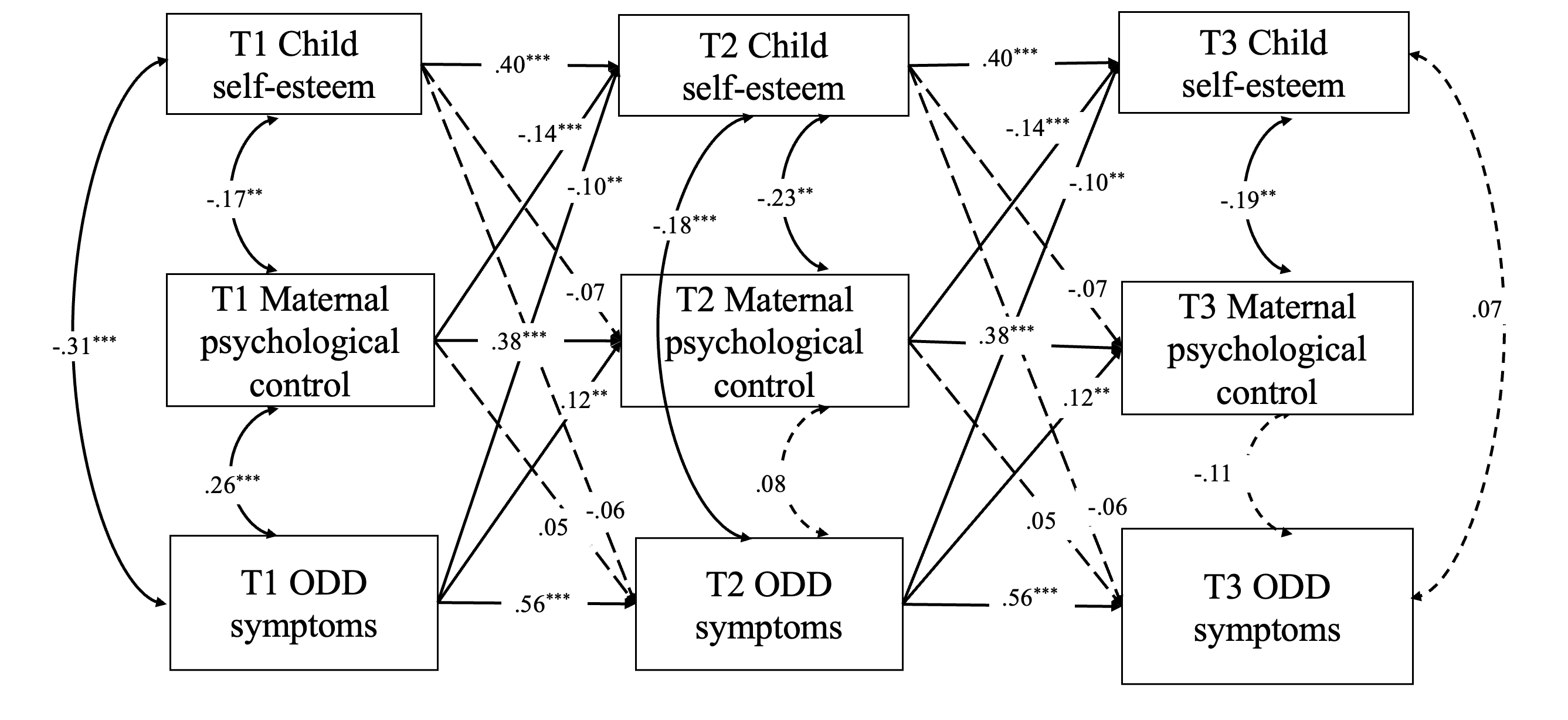
later, β = 0.12, *p* = .004.

*Figure S3.* Longitudinal associations between paternal psychological control, children’s self-esteem and ODD symptoms across three time points (i.e., two years).

**Paternal Model**

The initial unconstrained cross-lagged panel model showed good model fit, CFI = 0.99, TLI = 0.94, RMSEA = .06, SRMR = .02^[[2]](#footnote-2)^. Next, we constrained autoregressive and cross-lagged paths to be equal from T1 to T2 and from T2 to T3. The time-constrained model showed a similar model fit as the time-unconstrained model, CFI = 0.99, TLI = 0.98, RMSEA =.03, SRMR = .03; Δχ²= 5.48, *p* =.790. Thus, these paths did not significantly differ across time. As such, we interpreted findings from the time-constrained model (see Figure S4).

Concurrent associations indicated that higher levels of maternal psychological control were associated with lower levels of self-esteem at each measurement time, *r*s < -.14, *p*s < .007. Higher levels of ODD symptoms were associated with lower levels of self-esteem at T1 and T2, *r*s < -.17, *p*s < .002. Higher levels of ODD symptoms were associated with higher levels of maternal psychological control at T1, *r* = .22, *p* < .001. Autoregressive effects indicated that both maternal psychological control, children’s self-esteem and ODD symptoms showed considerable rank-order stability, βs > 0.34, *p*s < .001. Cross-lagged effects indicated that, higher levels of paternal psychological control predicted lower levels of child self-esteem, β = -0.08, *p* = .002, and higher levels of child ODD symptom one year later, β = 0.07, *p* = .003. Higher levels of child ODD symptom predicted lower levels of child self-esteem one year later, β = -0.12, *p* = .001, and higher levels of maternal psychological control one year later, β = 0.13, *p* = .001.


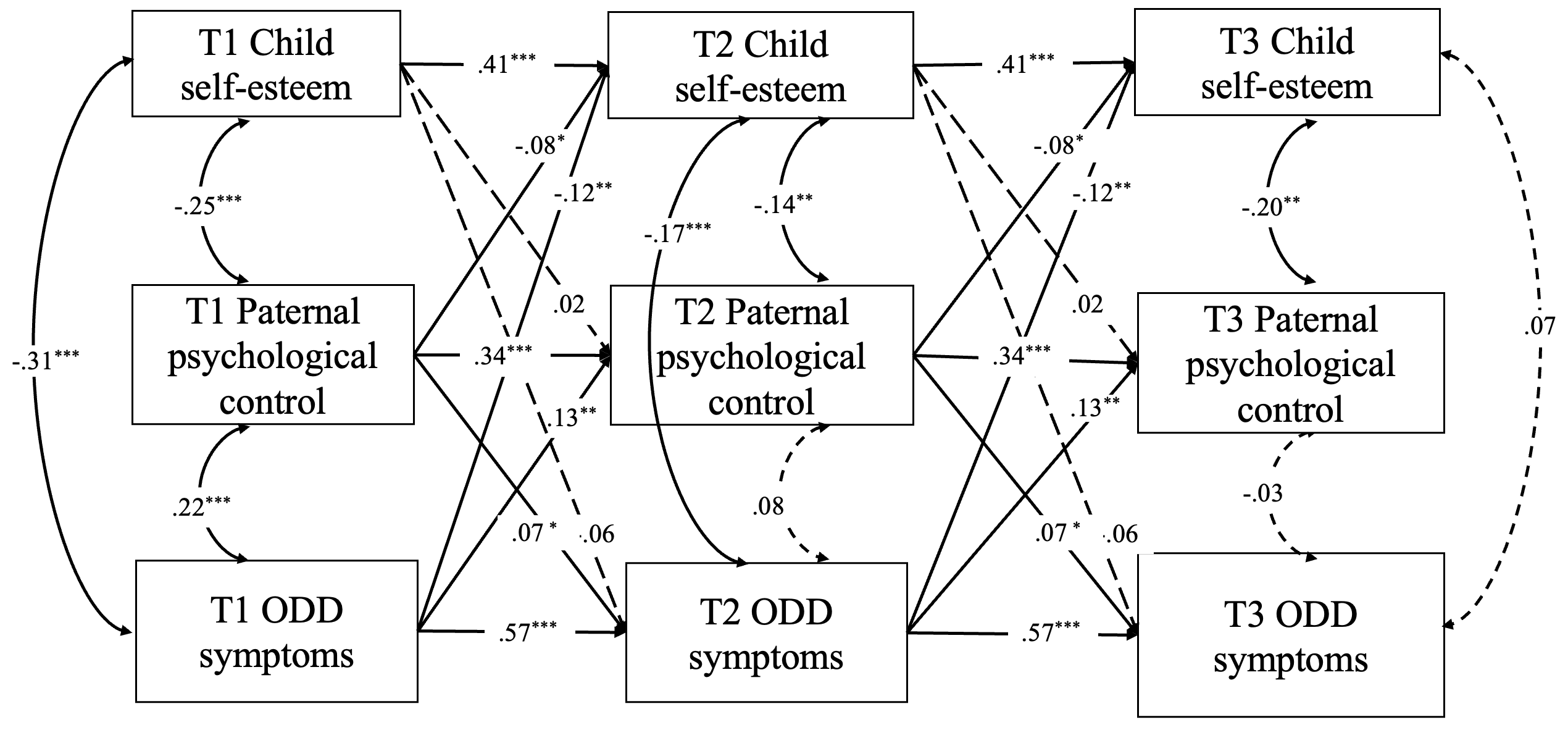


*Figure S4.* Longitudinal associations between paternal psychological control, children’s self-esteem and ODD symptoms across three time points (i.e., two years).

1. Following model fit indices, we added the autoregressive paths of T3 regressed on T1 to enhance the model fit [↑](#footnote-ref-1)
2. Following model fit indices, we added the autoregressive paths of T3 regressed on T1 to enhance the model fit [↑](#footnote-ref-2)
